# Unravelling Protein–Fungal Hyphae Interactions at the Nanoscale

**DOI:** 10.1021/acsami.5c01064

**Published:** 2025-04-17

**Authors:** Mary C. Okeudo-Cogan, Brent S. Murray, Rammile Ettelaie, Simon D. Connell, Michelle Peckham, Ruth E. Hughes, Martin J. G. Fuller, Stewart J. Radford, Anwesha Sarkar

**Affiliations:** † School of Chemical and Process Engineering, 150405 University of Leeds, Leeds LS2 9JT, U.K.; ‡ Food Colloids and Bioprocessing Group, School of Food Science and Nutrition, University of Leeds, Leeds LS2 9JT, U.K.; § School of Physics and Astronomy, University of Leeds, Leeds LS2 9JT, U.K.; ∥ Faculty of Biological Sciences, University of Leeds, Leeds LS2 9JT, U.K.; ⊥ Quorn Foods, Station Road, Stokesley, North Yorkshire TS9 7AB, U.K.

**Keywords:** self-consistent field calculations, colloidal interactions, protein adsorption, meat analogues, DLVO, STED, AFM

## Abstract

Fungal hyphae have demonstrated their importance in developing environmentally friendly, multiscale, composite assemblies where animal-derived proteins have been predominantly used as binders. Now, an ongoing challenge is to replace those high-performance animal protein binders with ecofriendly, plant-based alternatives. While the majority of studies have focused on the binding implied by rheological observations, relatively little is known about how such animal proteins bind to hyphal surfaces at nanometric length scales, and this knowledge is required to replace animal-derived binders with plant protein alternatives. Here, we decode intermolecular interactions of plant protein-based binders such as potato protein (*PoP)* to fungal (Fusarium venenatum *)* hyphae in comparison to a classic animal protein-based binder (egg white protein, *EWP*) using a suite of theoretical and experimental approaches. Self-consistent field calculations modeling fungal hyphae as weakly hydrophobic, parallel cylinders predicted differences in the interaction potentials between the model protein layers, showing that *EWP* had an attractive potential across a broad range of conditions, in contrast to *PoP* that was mainly repulsive. Stimulated emission depletion (STED) microscopy of protein-coated fungal hyphae confirmed that *EWP* delivers a uniform and complete coverage, while *PoP* naturally aggregates, resulting in more patchy coverage. Experimental interaction forces were measured using colloidal probe atomic force microscopy, confirming the influence of non-Coulombic forces particularly dominating in *PoP*, and attractive forces in *EWP*, further differentiating their respective binding mechanisms. Collectively, this multimethodological study provides a first-hand molecular explanation of the weaker hyphal-binding properties of aggregated plant proteins at the nanoscale, consistent with the previously reported macroscale observations.

## Graphical abstract

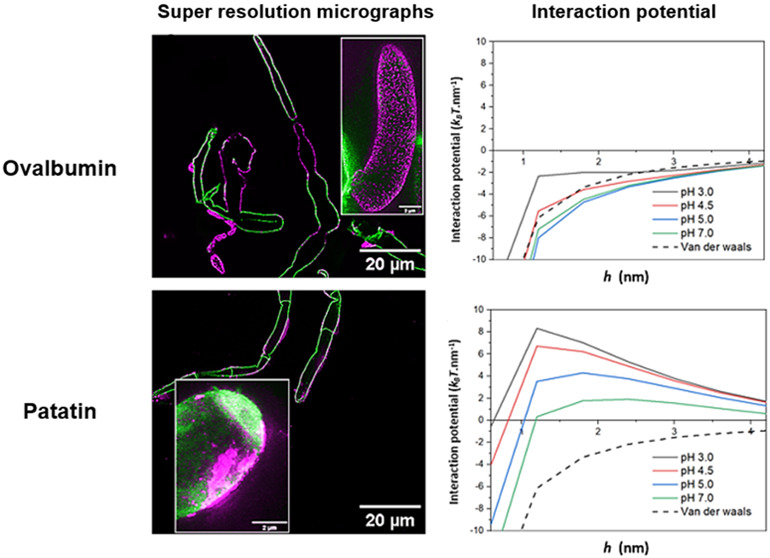

## Introduction

Microorganisms have become a valuable sustainable resource leading the advancement toward greener global material applications across a wide range of fields such as energy, agriculture, food, packaging, pollution, and chemical industries.^1–3^ Specifically in food and feed applications, the sharp rise in whole microbial biomass commonly known as ‘single-cell’ proteins as new protein sources is driven in part due to the demand for sustainable protein assemblies.^4^ One such single-cell protein is the filamentous fungus Fusarium venenatum referred to as ‘mycoprotein’, is the main ingredient in a popular composite assembly; in other words, an alternative meat product, for over four decades.^5,6^ Mycoprotein has been shown to have a significantly lower water and carbon footprint when compared to traditional animal meat.^7^ In addition, its hyphal structure adds a material advantage in the creation of meat-like textural properties reducing the need for advanced texturizing technologies required by most alternative nonanimal-sourced proteins.^5,6^

Utilization of the filamentous structures of fungal hyphae for fabricating composite macroscopic assemblies necessitates the addition of protein binders. Food binders are materials that facilitate and/or magnify the interaction of components through mechanical, adhesive, or chemical means to form a cohesive heterogeneous matrix with desired structural properties.^8^ Traditionally, egg white protein (*EWP*) has been used as a classic binder of fungal hyphae and has been shown to give desirable textural properties.^5,6^ However, increasing demand for ecofriendly alternatives to animal protein-sourced binders offer plant proteins such as potato protein (*PoP*) as suitable replacements.^6,9^ Earlier studies have shown that although the main protein in *EWP* and *PoP* have similar properties such as their molecular weight, isoelectric point, and solubility profile, the *EWP* composite has more desirable macroscopically determined mechanical properties when compared to the *PoP*-derived composite.^6,7,9,10^ However, the fundamental reasons for the superior performance of *EWP* over plant proteins are not understood, and without this knowledge, rational progress in animal-free formulation cannot be made.

Herein, we decode the underlying mechanism of interaction of these proteins with the hypha surface using a combination of experimental and theoretical techniques. We explore the adsorption of pure ovalbumin and patatin, making up more than 40–50% of the total protein in each case to the hyphal surface via the numerical Scheutjens–Fleer self-consistent field (SCF) theory.^11–15^ We hypothesize that there are significant differences in the surface organization of both these proteins at the hyphal surface at length scales far below the diffraction limit of optical microscopy, which were therefore not resolved by earlier confocal microscopic studies.^6,9^ Theoretical SCF predictions are supported with super-resolution stimulated emission depletion (STED) microscopy and resin-embedded transmission electron microscopy (TEM) of the fungi hyphae and protein composite to resolve at the nanoscale the differences between the binding behavior of *EWP* and *PoP* onto the hyphal surface. Direct experimental evidence of the interaction forces between surfaces coated with layers of the *EWP* and *PoP* were provided experimentally via colloidal probe atomic force microscopy (AFM) force–distance (FD) curves. By taking into consideration the theoretical along with qualitative and quantitative experimental data, we draw a complete picture of the underlying molecular mechanisms explaining the superior performance of *EWP* binding to fungal hyphae, in comparison with *PoP*.

## Experimental Section

### Materials

Egg white protein (*EWP*), Solanic 200 potato protein (*PoP*), and chilled heat-treated fungi hyphae paste at ∼24 wt % solids were supplied by Quorn Foods (Stokesley, North Yorkshire, U.K.). Type I (Milli-Q) water (Millipore, Bedford, U.K.), with a minimum resistivity of 18.2 MΩ cm and analytical grade chemicals were used in the preparation of all samples unless otherwise specified.

### Surface Charge of Fungal Hyphae

The electrophoretic mobility of the hyphae as a function of pH can give an indication of the surface charge of the fungal hyphae. The fungal paste was frozen in liquid nitrogen and homogenized in a ceramic mortar and pestle. This process was repeated 4 times until a fine powder was obtained. The fine powder was dispersed in Milli-Q water and centrifuged at 1000*g* for 10 min twice to remove cellular debris. After the supernatant was decanted, the powdered paste was resuspended in Milli-Q water at 0.5 g/L and filtered through 0.2 μm asymmetric polyether sulfone membrane (ThermoFisher Scientific, Loughborough, U.K.) to remove larger fragments. The resulting filtrate was adjusted to various pH (pH 3–7), and electrophoretic mobility and ζ-potential values were recorded using DTS1070 folded capillary electrophoresis cells in a Malvern Zetasizer Ultra (Malvern instruments Ltd., Worcestershire, U.K.) at 25 °C. This was repeated three times for each pH condition.

### Theoretical Modeling of Protein Interaction

#### SCF Calculations

Scheutjens–Fleer self-consistent field theory (SCF) is a numerical tool used to predict theoretically, self-assembly, adsorption, and interaction potential of complex heterogeneous systems, consisting of solvent, electrolyte, and polymers (protein) at equilibrium. Here, it is applied to dispersed proteins (*EWP* or *PoP*) across a gap between two planar surfaces whose separation distances are discretized into individual layers each consisting of a regular cubic lattice.^11,14,15^ The discretization is the consequence of the numerical nature of these calculations. Each species occupies a unit cell (assigned here to have the nominal size of 0.3 nm, which is roughly the length of a peptide bond) with the interaction experienced by each residue within each layer assuming the Bragg–Williams approximation of random mixing. In other words, the abundance of other residues, solvent, and ions around any monomer is taken as being the same as that in an entire layer. The most probable state is determined as the one that minimizes the free energy of the system. That is to say the density profile distribution for each species, with the variations perpendicular to the surface across the gap, which yield the lowest free energy.^11^

In this work, the model system consists of four main components: (i) solvent—water, (ii) protein—ovalbumin (representing *EWP*) or patatin (representing *PoP*), plus the (iii) monovalent salt NaCl divided into positive (Na^+^) and negative (Cl^–^) ions, and the (iv) hyphal surface. The electrophoretic mobility measurements in Table S2 indicate that the fungal hyphae are negatively charged across the whole experimental window of pH tested (3.0 to 7.0), although the absolute magnitude increases as the pH rises. Here, we use these measurements to confirm that the model hyphal surface should be assigned a somewhat lower degree of hydrophobicity of −1.0 *k*_B_ *T* than the more commonly encountered −2.0 *k*_B_ *T* Flory–Huggins χ interaction between the surface and hydrophobic amino acids residues in SCF calculations of this kind.^11–13,16,17^

SCF calculations were performed at a range of pH and [NaCl], relevant to real processing conditions of the fungal–protein composite and in line with previous experimental work: pH 3.0, 4.5, 5.0, and 7.0 with background NaCl volume fractions *ϕ*_s_ = 0.001, 0.01, and 0.05, which are approximately equivalent to [NaCl] = 10, 100, and 500 mM.^6,9^

#### Protein Models

The primary structures of ovalbumin (P01012) https://www.uniprot.org/uniprotkb/P01012/entry and patatin (P07745) https://www.uniprot.org/uniprotkb/P07745/entry were obtained from the protein database UniProt.^18–20^ Ovalbumin, the major fraction in *EWP* is a phosphoglycoprotein with 386 amino acids.^21^ Ovalbumin has an oligosaccharide side chain comprising of a 5:2 ratio of mannose to *N*-acetyl glucosamine (GlcNAc) with structure of α -d-Manp-(1→6)-[α-d-Manp-(1→3)]-α-d-Manp-(1→6)-[α-d-Manp-(1→3)]-β-d-Manp-(1→4)-β-d-GlcNAcp-(1→4)-β-d-GlcNAcp→Asn^292^ linked to asparagine (Asn)^292^.^22^ Patatin, the major protein in *PoP*, has 363 amino acids, excluding the first 23 amino acids that are the signal molecules and not part of the patatin chain.^20^ Patatin has a more complex glycosylation pattern, with its primary structure glycosylated at Asn^60^, Asn^90^, Asn^115^, and Asn^202^ (no glycan) with oligosaccharides consisting of xylose (Xyl), fucose (Fuc), mannose (Man), and *N*-acetyl glucosamine at a ratio 1:1:3:2.^23,24^ For simplicity, the side chain α-d-Manp-(1→3)-[α-d-Manp-(1→6)]-[β-d-Xyl-(1→2)]-β-d-Manp-(1→4)-β-GlcNAc-(1→3)-[α-l-Fuc-(1→3)]-GlcNAc-Asn^115^ at Asn^115^ (UniProt) was used in the calculations (Figure 2).

The amino acids were divided into six groups; group 1—hydrophobic, group 2—polar non-charged, group 3—positively charged, group 4—histidine (distinct p*K*_a_ compared to other positively charged amino acids), group 5—negatively charged, and group 6—phosphoserine (phosphorylated serine in just ovalbumin) based on their p*K*_a_, charge at neutral pH, and degree of hydrophobicity.^11–13,16,17^ The carbohydrate side chain of both proteins was modeled as uncharged hydrophilic monomers, represented as group 7.^11^ The solvents Na^+^ and Cl^–^ were assigned groups 0, 8, and 9, respectively. The short-range interactions between each species, those with the solvent and interactions with the surface, are represented via the Flory–Huggins χ parameter detailed in Table 1, in accordance with previous work in the literature, with modification of the surface-hydrophobic residue (group 1) interaction parameter to represent the slightly less hydrophobic nature of the hyphae surface here.^11,12^

**Table 1:** Flory–Huggins *χ* Interaction Parameters (in units of *k*_B_ *T*) Assigned to Each Monomer Group, Positive and Negative Salt Ions, Surface, and *pK*_*a*_ Values for Each Ionizable Group^11,12^

monomer type	0	1	2	3	4	5	6	7	8
0—solvent	0	1.0	0	0	0	0	0	–1.0	–1.0
1—hydrophobic residues	1.0	0	2.0	2.5	2.5	2.5	2.5	2.5	2.5
2—polar residues	0	2.0	0	0	0	0	0	0	0
3—positive residues	0	2.5	0	0	0	0	0	0	0
4—histidine (His)	0	2.5	0	0	0	0	0	0	0
5—negative residues	0	2.5	0	0	0	0	0	0	0
6—phosphoserine (Pser)	0	2.5	0	0	0	0	0	0	0
7—carbohydrate groups	0	2.5	0	0	0	0	0	0	0
8—Ion (+)	–1.0	2.5	0	0	0	0	0	0	0
9—Ion (−)	–1.0	2.5	0	0	0	0	0	0	0
surface	0	–1.0	0	0	0	0	0	0	0
p*K*_a1_	–	–	–	10	6.75	4.5	3	–	–
p*K*_a2_	–	–	–	–	–	–	7	–	–

#### Interaction Potential between Two Parallel Cylinders

The interaction potential, obtained as the change in free energy of two planar surfaces when at a distance *r* compared to when infinitely apart, is plotted in units of *k*_B_ *T*.a_0_^–2^ in Figure S4. This information can be converted to the interaction potential between two parallel cylinders which more approperiately reflect an idealized model of fungal hyphae. We modeled the hyphae as smooth cylinders with a uniform cross-sectional diameter of 3 μm,^5,6^ assumed to be lying parallel to each other. Noteworthy, this necessitated a more complex application of Derjaguin approximation to SCF-calculated interaction potential between planar surfaces, which is not commonly given in the literature. Crossed cylinders (i.e., at right angles) are of course covered in the classic case of the surface force apparatus, and the overall interaction will be much weaker.^25,26^ Most observations of the microstructure of mycoprotein pastes show the majority of the fibers lying approximately parallel to each other, which is indeed what imparts the uniques texture of the material.^6,9^

Framing the problem of the mathematical derivation of interaction force *f*_cy_(*h*) between two parallel cylinders of equal radius (*R*) from forces operating between two planar surfaces *f*_pl_(*h*) via the Derjaguin approximation is shown below in eqs 1–7, where *h* is surface to surface separation between the cylinders. The separation distance between two segments of surface of size *Rdθ*, residing opposite each other at a polar angle θ, as illustrated in Figure 1 is obtained by noticing first that $x\approx R\frac{\sin^{2}\theta }{2}$ for angles sufficiently close to zero, i.e., close to where the closest approach of the two surfaces occurs. The separation between these two segments of the surface is then *h* + 2*x* = *h* + *R* sin^2^ θ.

**Figure 1:**
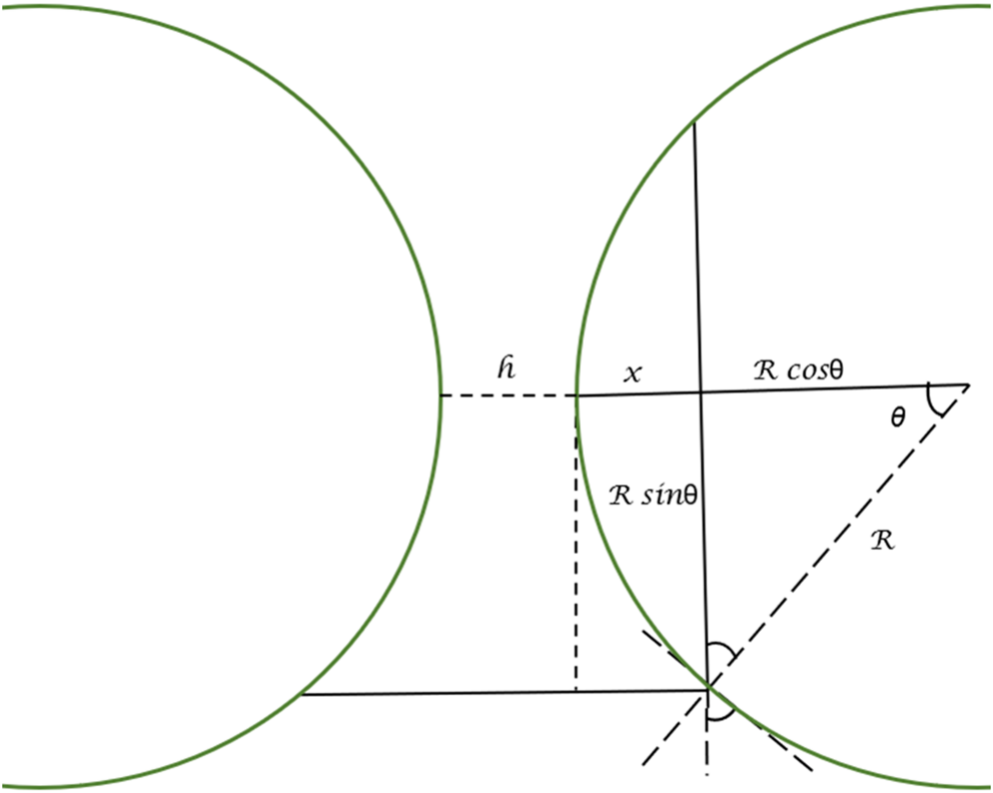
Schematic representation of the parallel cylinder geometry used to represent the hyphal interactions. Where *h* is the separation distance between the parallel cylinders, and *R* is the radius of the cylinder.

Summing up the force contribution from all such surface segments, we have

1 $$f_{\mathrm{cy}}\left(h\right)=\int_{0}^{\pi /2}2f_{\mathrm{pl}}\left(h+R\;\sin^{2}\theta \right)\cdot R\;\cos\theta \;d\theta$$

where the surface elements are resolved in the parallel direction facing each other, *i.e., R* cos­(θ)­d*θ.* Now making the following change of the integration variable.

2 $$R\;\sin^{2}\theta +h=y$$

and therefore,

3 $$2R\;\cos\theta \;d\theta =\frac{dy}{\sin\theta }=\frac{dy\sqrt{R}}{\sqrt{\left(y-h\right)}}$$

the integral in eq 1 becomes

4 $$f_{\mathrm{cy}}\left(h\right)=\sqrt{R}\int_{h}^{\infty }\frac{f_{\mathrm{pl}}\left(y\right)}{\sqrt{y-h}}dy$$

The interaction potential between the cylinders at a separation distance of *r* is thus

5 $$V_{\mathrm{cy}}\left(r\right)=\int_{r}^{\infty }f_{\mathrm{cy}}\left(h\right)dh=\sqrt{R}\int_{r}^{\infty }\int_{h}^{\infty }\frac{f_{\mathrm{pl}}\left(y\right)}{\sqrt{y-h}}dydh$$

The SCF calculations thus provide us with the numerical values of the interaction potential between two flat surfaces, namely, *V*_pl_(*y*), rather than the force *f*_pl_(*y*). Therefore, it is also more convenient to express eq 5 in terms of the former, if possible. To do so, we alter the order of integration in eq 5 to obtain

6 $$V_{\mathrm{cy}}\left(r\right)=\sqrt{R}\int_{r}^{\infty }f_{\mathrm{pl}}\left(y\right)\int_{r}^{y}\frac{1}{\sqrt{y-h}}dhdy=2\sqrt{R}\int_{r}^{\infty }\sqrt{y-r}f_{\mathrm{pl}}\left(y\right)dy$$

Finally, recalling that *f*_pl_(*y*) = −*dV*_*pl*_(*y*)/*dy* and performing an integration by parts in eq 6, we arrive at the required result:

7 $$V_{\mathrm{cy}}\left(r\right)=\sqrt{R}\int_{r}^{\infty }\frac{V_{\mathrm{pl}}\left(y\right)}{\sqrt{y-r}}dy$$

providing *V*_cy_(*r*) for two parallel cylinders in terms of *V*_pl_(*r*). Equation 7 applies to all cases generally, as long as *V*_pl_(*r*) drops faster than 1/√*r*, a condition that would be satisfied in any case as one of the requirements for the application of the Derjaguin approximation.

#### Interactions between Two Cylinders

The interaction *V*_cy_(*r*) between the two parallel cylinders modeling two fungal hyphae was calculated from the SCF-calculated interaction potential of planar surfaces *V*_pl_(*r*) via the Derjaguin approximation, as outlined above. If an analytical expression is available for *V*_pl_(*r*), then eq 7 may be evaluated to obtain a closed form equation for *V*_cy_(*r*). Herein, however, only numerical values of *V*_pl_(*r*) at certain discrete values of “*r*” are generated by our SCF calculations. Hence, the integral in eq 7 must be evaluated numerically. In carrying out this task, some care must be exercised, particularly in dealing with the integrand at points close to *y* = *r* where the integrand diverges due to the presence of $1/\sqrt{(y-r})$ term.

### Transmission Electron Microscopy

#### Sample Preparation and Resin Embedding

Fungal hyphae at 20 wt % solids (*MYC*), *MYC* with 3 wt % *EWP* (*MYC-EWP*), and *MYC* with 3 wt % *PoP* (*MYC-PoP*) were prepared in Milli-Q water. Each sample was dehydrated using an ascending alcohol series of 20, 40, 60, 80, and two 100% ethanol with 1 h incubation time. The dehydrated samples were incubated in 100% propylene oxide twice for 20 min. Thereafter, they were incubated in a 1:1 propylene oxide—Araldite solution for 4 h, and 1:3 propylene oxide—Araldite mixture for 4 h, 100% Araldite for 6 h and then polymerized in fresh 100% Araldite at 60 °C overnight.^27^ Each sample was sectioned using a Reichert–Jung Ultracut-E ultramicrotome to ca. 90 nm thickness. The sections were placed on 3.05 mm grids and stained with Reynolds lead citrate for 20 min.^28^

#### Transmission Electron Microscopy and Image Processing

Images were captured on a Gatan UltraScan 4000 CCD on FEI Technai G2 Spirit TEM (ThermoFisher Scientific, Loughborough, U.K.) at 120 kV using Digital microscope software. The images were processed by using Fiji software.

### Stimulated Emission Depletion (STED) Imaging

#### Composite Preparation

A solution of 1.0 g/L of each protein, *EWP* and *PoP*, in Milli-Q water was prepared to which 0.1 g/L *MYC* was added. Both mixtures were incubated at room temperature for 1 h to allow the proteins to coat the hyphal surface. The hyphae were then washed twice in phosphate buffer saline (PBS) through a 0.22 μm nylon syringe filter. The protein-coated hyphae were recovered with a filter back-wash. Experimental controls of protein solution and fungal hyphae dispersions alone at identical concentrations were prepared alongside the washing steps omitted.

#### Immobilisation and Surface Blocking

About 50 μL of each composite solution was placed onto clean poly-l-lysine-coated No.1.5 coverslips and incubated overnight at 4 °C under enclosed humid conditions to prevent the slides from drying out. The coverslips were washed twice with PBS, each wash step taking 5 min. And 50 μL of 5% bovine serum albumin (BSA) in PBS was added to each coverslip and incubated at room temperature for 30 min to prevent nonspecific antibody binding. The slides were then washed 3 times in PBS.

#### Immunolabeling

Both 50 μL of polyclonal antipatatin (Agrisera, Vännäs, Sweden) and antiovalbumin (Bio-Rad, Hertfordshire, U.K.) raised in rabbits and diluted 1:50 into 5% BSA/PBS solution were added to each composite coverslip, respectively, and incubated for 2 h at room temperature. The coverslips were then washed 3 times in PBS. Around 50 μL portion of the secondary antibody, goat anti-rabbit IgG Star Red (Abberrior, Gottingen, Germany) diluted 1 in 100 into 5% BSA/PBS solution was added and incubated in the dark at room temperature for 1 h. The slides were then washed 3 times in 1× PBS. The slides were counter stained with 50 μL of 20 μg/mL Alexa Fluor 594 conjugated to wheat germ agglutinin (Invitrogen, ThermoFisher Scientific, Loughborough, U.K.) in 1× PBS for 20 min. The coverslips were washed twice in 1× PBS and blotted dry. And 5 μL of mount Prolong Gold (ThermoFisher Scientific, Loughborough, U.K.) was added to prevent fluorescence fading, and the coverslips were mounted onto glass slides. The slides were cured in the dark at room temperature for 24 h before imaging.

#### STED Imaging and Processing

2D or *z*-stack images were acquired on a STEDYCON system (Abberior Instruments, Göttingen, Germany) using a depletion 775 nm laser. The STEDYCON is attached to a Zeiss Axio-observer Z1 microscope, and STED images were acquired using a Leica 100x/1.4 oil immersion objective. Excitation channel and emission detection wavelengths of 640 and 660 nm for Star Red and 561 and 618 nm for Alexa Fluor 594 dye, respectively, with the detection gated window starting and ending at 1 and 7 ns. Images were deconvolved using the automated deconvolution express settings in the Huygens software (Scientific Volumetric Imaging, Netherlands; version 23.04) to obtain more detailed images. Further processing to highlight areas of interest and include scale bars was performed using Fiji software.^29^

### Atomic Force Microscopy—Colloid Probe Force Measurements

#### Sample Preparation

Protein solutions of 1.0 wt % *EWP* and *PoP* were prepared in Milli-Q water at room temperature. Milli-Q water was adjusted to pH 3.0, 4.5, 5.0, and 7.0, respectively, by adding very little amounts of 1 M HCl or NaOH and used to adjust the pH of proteins adsorbed to silicon surfaces.

#### Atomic Force Microscopy (AFM)

All measurements were carried out using Bruker Multimode8 AFM (Massachusetts, USA) equipped with a Bruker Nanoscope V controller (Massachusetts, USA). A silicon dioxide (SiO_2_) spherical colloidal probe sQube CP_CONT-SiO-A-5 (NanoAndMore, Karlsruhe, Germany) of radius 1.0 μm attached to an uncoated silicon AFM cantilever (force constant 0.2 N/m, resonance frequency 13 kHz) was used for all measurements. Once the deflection sensitivity had been calibrated on clean silicon, the spring constant of each probe was obtained before measurement using the thermal noise method in air with values in the range of 0.28 ± 0.02 N/m obtained. All measurements were carried out using a fluid cell. Precut silicon wafer was prepared as single use substrates. The silicon was simply rinsed with Milli-Q water and dried with an N_2_ gas gun prior to use to remove the Si fragments created when cutting. About 100 μL of each protein was deposited on the silicon wafer and allowed to adsorb onto the silicon wafer and the colloidal probe for 1 h at room temperature (ca. 21 °C). Excess nonadsorbed proteins were washed out by passing 1 mL of Milli-Q water through the fluid cell three times. Then, 1 mL of pH-adjusted water was passed through the fluid cell and left for 10 min to modify the pH of the adsorbed protein layer working from pH 7.0 to pH 3.0.

#### Force Volume Measurements and Force–Separation Distance (*F*–*h*) Curves

Force volume maps were acquired to ensure that each curve was measured in a different location and moving systematically across a wide area. Around 1024 force volume curves were recorded for each 10 μm force map at a resolution of 32, a scan rate of 0.997 Hz, a forward and reverse tip velocity of 1 μm/s, a ramp size of 100 nm, and a trigger threshold set at 100 nm. For each protein at a pH point, measurements in triplicates with two repeats were carried out (*n* = 2 × 3). About 50 of the extend force–distance curves were analyzed, and 6 curves were plotted for each protein—pH condition, highlighting protein repulsion as a function of pH. The extracted *F*–*h* curves were plotted using OriginPro version 2019b.

#### Van Der Waals Interaction

The van der Waals interaction was calculated using the model of Wang, Wang, Hampton, and Nguyen^30^ for a silica sphere interacting with a silicon substrate, as in our experiments. The effective Hamaker constant *A*_H_ fitted to Wang, Wang, Hampton, and Nguyen^30^ AFM data at similarly low ionic strength in a 1:1 electrolyte was 2.3 × 10^–21^ J. This is the value of *A*_H_ we have used here. We have also assumed a background electrolyte concentration of 10^–4^ M for pH 4.5 and pH 7.0 but increased this to 10^–3^ M for pH 3.0, to take into account the concentration of H^+^ (note that there was no other added buffer or salts added to the experimental systems).

### ζ-Potential and Hydrodynamic Diameter Measurements

The ζ-potentials and hydrodynamic diameters of 0.3 wt % *EWP* (refractive index = 1.51, absorption = 0.001) and *PoP* (refractive index = 1.45, absorption = 0.001) solutions were measured in triplicate using Malvern Zetasizer Ultra (Malvern instruments Ltd., Worcestershire, U.K.). Both proteins were dissolved in Milli-Q water (dispersant: refractive index = 1.33, viscosity = 0.8872 cP, dielectric constant = 78.5) and adjusted to pH values between 7.0 and 3.0 using 1 M HCl or 1 M NaOH. The prepared solutions were filtered through 0.22 μm nylon syringe filter and placed in a DTS1070 cell and DTS0012 disposable cuvettes (PMMA, Wertheim, Germany) for ζ-potential and particle size measurements, respectively. Measurements were taken at 25 °C using backscattered light at 173° detection angle with samples left to equilibrate for 120 s.

### Statistical Analysis

All means and standard deviations were calculated for triplicate measurements (*n* = 3 × 3). One-way analysis of variance (ANOVA) and posthoc Tukey tests were used to calculate mean separation at a 5% level of significance using Minitab 21 software.

## Results and Discussion

### Self-Consistent Field (SCF) Calculations of Coverage and Interactions

For SCF calculations, we modeled the fungal hyphae as parallel cylinders of radius of 1.5 μm, possessing a mildly hydrophobic surface due to the hyphal surface negative charge as a function of pH estimated from ζ-potential measurement of crushed fungal hyphae (see the Supporting Information file, Table S2).^6^ Interactions between this surface and the hydrophobic amino acid residues were assigned a favorable Flory–Huggins χ parameter of −1.0 *k*_B_ *T* (see Experimental Methods, Table 1), typical of such hydrophobic interactions.^11,12,31^ The bulk protein volume fraction was set at 1.0 × 10^–7^ equivalent to ∼1.35 × 10^–5^ wt % assuming average protein density of 13.5 μg/mL.^32^ We chose low bulk values to reflect the expectation that most of the protein will be adsorbed and not be present in bulk solution. However, it is important to note that this does not mean that the amount of protein in the system is low. As for all other SCF calculations of protein adsorption, ovalbumin and patatin were modeled as unfolded primary chains with amino acids divided into six groups based on their p*K*_a_, charge at neutral pH, and degree of hydrophobicity shown in Figure 2.^11–13,16,17^ Due to the necessary simplification of the amino acid classification, the theoretical isoelectric points (pI) of ovalbumin and patatin were calculated as 5.30 and 5.87, respectively (Supporting Information file, Figure S1). This compares favorably with experimental pI values of 4.5 and 4.5–5.1, respectively, determined from electrophoretic mobility measurements of the native proteins measured using a laser Doppler velocimetry (ζ-potential) (Table S1).^9^

**Figure 2:**
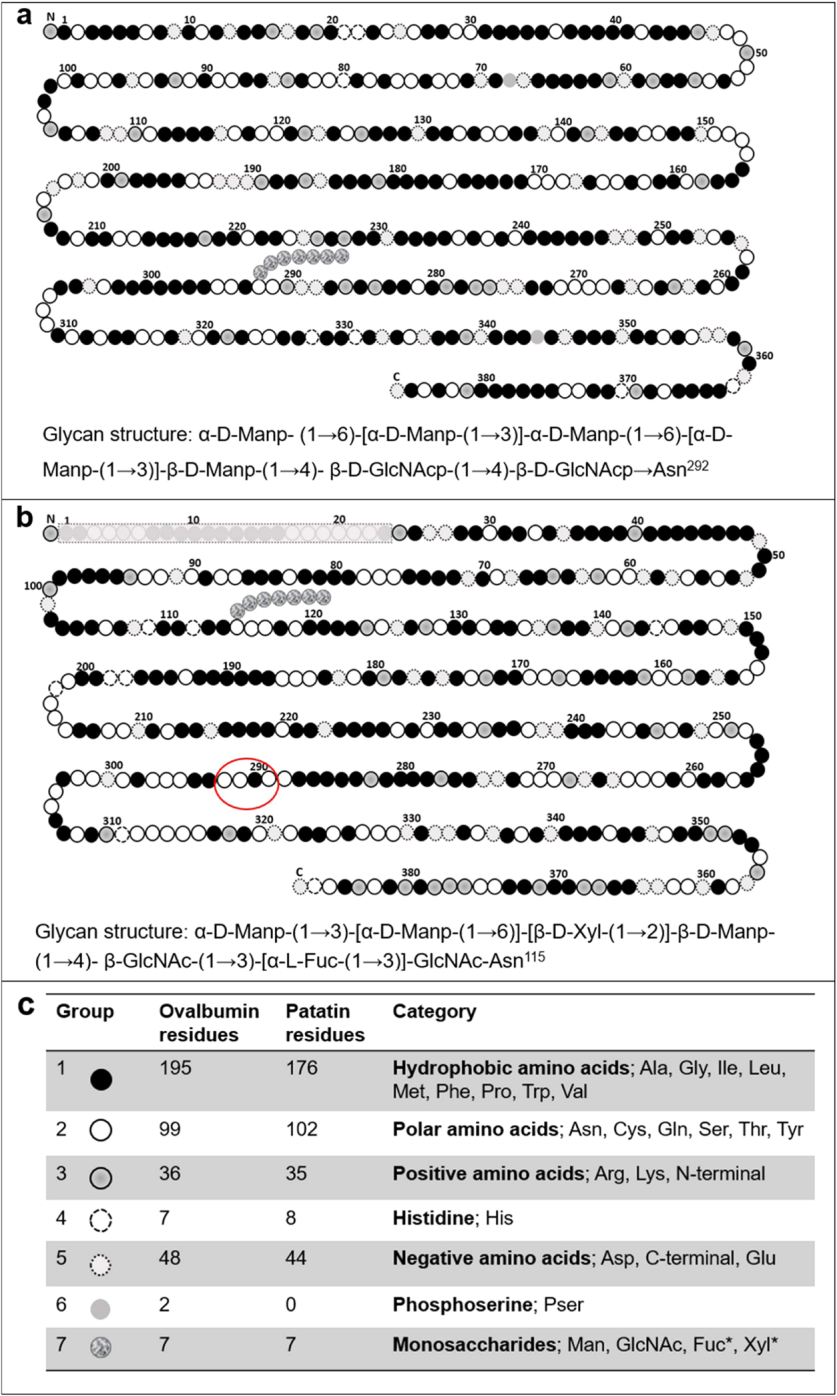
Linear amino acid sequence of model proteins taken from UniProt protein database used in our SCF calculations.^18–20^ Schematic illustrations of the primary sequence of (a) ovalbumin representing *EWP* and (b) patatin representing *PoP*, with the amino acid residues classified (c) into six groups. The seventh group represents the carbohydrate side chain with detailed glycan structure units attached to both proteins. Patatin’s signal chain 1–23 amino acids are grayed out and was not included in the models. The red circle in (b) indicates the start of patatin’s hydrophilic "tail".

First, we explore the adsorption characteristics of the proteins before moving on to how this adsorption affects the interactions between the hyphal surfaces. The parallel cylinder case is not a frequently explored geometry in this type of calculations in the literature (most calculations involve flat plates or spheres), but it is most relevant to the type of fiber composite present in the mycoprotein products of interest here. This therefore requires what we believe are some original transformations from the starting numerical SCF plate–plate interaction results to the parallel cylinder case by making use of the Derjaguin approximation.

### Protein Adsorption on the Fungal Hyphae Surface

For proteins to be effective binders, they have to adsorb to the surface and facilitate attractive interactions commonly measured as stickiness or adhesiveness, which otherwise have little or no interaction between them.^8^ Denatured proteins have increased affinity for hydrophobic surfaces due to their more exposed hydrophobic amino acids.^33^ Here, we consider the interfacial adsorption properties of ovalbumin and patatin modeled using their primary structure (Figure 2). This is obviously an approximation, but since globular proteins become more unfolded with adsorption (at least when there is plenty of surface available for adsorption at low initial bulk protein concentrations ∼1.35 × 10^–5^ wt %, as here), more of the primary structure does indeed become exposed to the adsorbing surface. Thus, this approximation has yielded results for many other globular proteins that seem to accord qualitatively with experimental measurements of their adsorption properties, recalling that the SCF methodology yields the equilibrium adsorbed state.^11,12,34,35^

SCF-calculated protein density profiles were plotted as the volume fraction of protein *ϕ*_p_ as a function of the perpendicular distance from the hyphal surface at the bulk protein volume concentration of ∼1.35 × 10^–5^ wt % (Figure S2). Ovalbumin (Figure S2a–c) and patatin (Figure S2d–f) are similar in their density profiles when they are bound to the hydrophobic surface. To illustrate the role of Coulombic forces, we calculated density profiles of the tested animal and plant proteins as a function of pH and ionic strengths. At the lowest background [NaCl] of 10 mM, the density profile of ovalbumin at the hydrophobic surface is highest (*ϕ*_p_ = 0.246) at the pH closest to the pI of pH 5.0 and declines as the protein becomes increasingly charged at pH values further away—the lowest *ϕ*_p_ (0.104) at the surface, in the range of pH values studied was observed at pH = 3.0 (Figure S2a). As expected, increasing the background [NaCl] to 100 mM (Figure S2b) and further to 500 mM (Figure S2c), caused charge screening effects dominate and narrow the differences in the profiles between the different pH values while simultaneously increasing the adsorbed amounts of ovalbumin: *ϕ*_p_ = 0.366 and 0.322 at pH 5.0 and pH 3.0 respectively, at 500 mM NaCl (Figure S2c).

Although we observed similar density profiles for patatin (Figure S2d–f), patatin’s adsorbed layer region extends farther away from the surface than ovalbumin at all measured conditions. For example, both proteins show a dense inner layer of ∼1 nm thickness, while for patatin, a less dense part of the layer extends to as far as ∼4.0 nm. For ovalbumin, this less dense, outer part is only ∼2.0 nm, with some slight variations depending on the [NaCl]. Thus, the adsorbed patatin molecules appear to stretch significantly further away from the surface, and this is due to the cluster of charged and/or polar amino acid residues found toward the C-terminus end of the chain (see Figure 2). To further illustrate the differences in the interfacial organization of ovalbumin and patatin, Figure 3 shows the average distance of each amino acid ranked from its N-terminus side, away from the hyphal surface.

**Figure 3:**
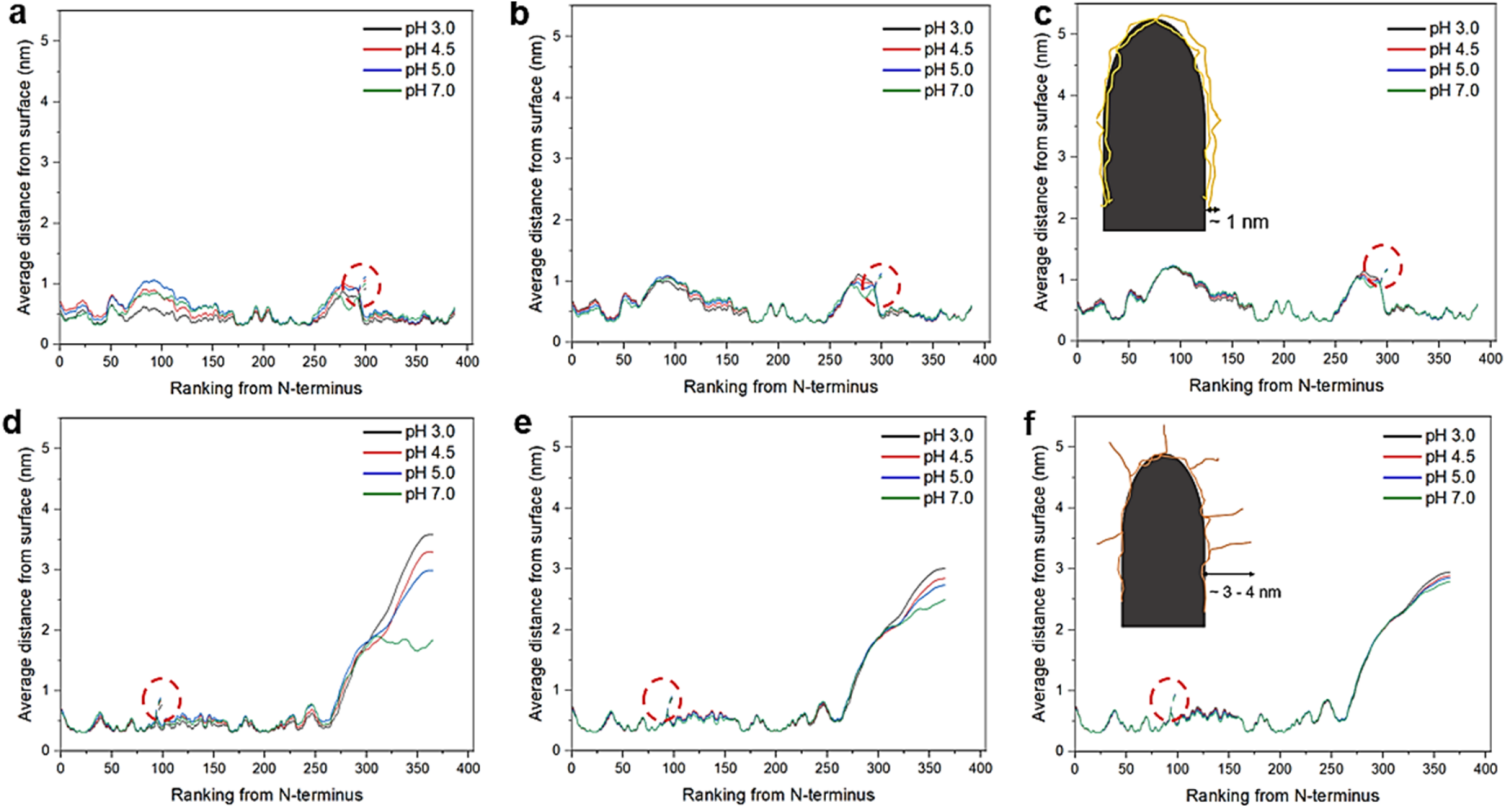
SCF-calculated distribution of amino acid residues on the fungal hyphae modeled as a weakly hydrophobic surface at bulk protein concentrations of ∼1.35 × 10^–5^ wt %. The average distance away from the surface of each amino acid residue, numbered consecutively from the *N*-terminus, of ovalbumin (a–c) and patatin (d–f) adsorbed onto a moderately hydrophobic surface, modeled using SCF calculations, plotted as a function of pH 3.0 (black solid line), 4.5 (red solid line), 5.0 (blue solid line), and 7.0 (green solid line) with carbohydrate side chain (dashed line, highlighted by the red dashed circle). Changes to the average distance as a function of salt volume fraction 0.001 (a, d), 0.01 (b, e), and 0.05 (c, f) are demonstrated for both proteins. A schematic illustration is shown highlighting the likely configurational differences of absorbed ovalbumin coating while patatin chains protruding in a patchy configuration on the surface are shown in (c) and (f), respectively.

At all measured [NaCl], ovalbumin lies very close to the surface, with amino acids 50–120 and 250–300 extending to ∼1 nm from the surface as a single protein interfacial layer, forming a roughly “M” shaped multiblock configuration. Not surprising, the slightly extended region has a higher ratio of polar and charged amino acids as well as the hydrophilic carbohydrate side chain (see Figure 2). Conformation of ovalbumin changes with pH at low [NaCl] (Figure 3a) but the variations are minimal at higher [NaCl] (Figure 3b,c), highlighting the importance of electrostatic repulsion for this protein. In contrast, patatin (Figure 3d–f) is divided into two regions forming more of a diblock type conformation, where the first block is hydrophobic and lies very close (<1 nm) to the surface while the second block is hydrophilic and extends away from the surface up to distances of ∼3–4 nm. Examination of the primary structure in Figure 2 shows that this outer part of the layer starts at the 288th residue from the N-terminus side (marked by a red circle) and forms a hydrophilic “tail” of 99 amino acids, 64 of which are charged or polar. This hydrophilic tail provides a “hairy” structure that should enhance electro-steric repulsion between hyphal surfaces coated in adsorbed patatin.

Adsorption isotherms of ovalbumin and patatin were calculated via the SCF scheme shown in Figure S3.^10,36,37^ Like most proteins, maximum surface coverage plateau occurs at very low concentrations, and that coverage remains largely unaltered by an increase in bulk concentration but showed sensitivity on pH and salt concentration. The highest and lowest adsorbed Γ were observed at pH 5.0 and pH 3.0, although at the highest [NaCl] the differences between pH 4.5, 5, and 7 are small (see Figures S3c and S3f), indicating that the charge of the protein only plays a secondary role, having largely been screened by the background electrolyte. This highest Γ observed at pH 5.0, at lower salt concentrations, was expected considering that pH 5.0 is closest to the pI, so mutual repulsion between the protein chains and the surface will be minimal, favoring adsorption. It should be noted that these values for Γ are all within the expected range for protein adsorption measured experimentally.^16,31^ Strikingly, Γ for patatin is 0.1 to 0.5 mg·m^–2^ higher than for ovalbumin under most conditions tested (Figure S3d–f). This is attributed to the somewhat diblock-like configuration adopted by patatin upon adsorption, where some of its amino acids at one end extend further away from the surface. It is well-known that a diblock polymer suffers a smaller configurational entropy penalty when adsorbed than those that lie flatter on the surface.^38^ With all of the above information being the same, this leads to a higher adsorption for patatin relative to ovalbumin.

### Super-Resolution Microscopy of Real Hyphae–Protein Composites

We now questioned how these predicted differences in protein adsorption and conformation are reflected in the organization of proteins at the surface of fungal hyphae with added *EWP* or *PoP* using super-resolution microscopy. Remarkably, STED micrographs clearly demonstrate that *EWP* coats the hyphae uniformly where protein–hyphae interaction dominates over any *EWP*–*EWP* aggregation (Figure 4a–c). In contrast, *PoP* formed a rather patchy attachment on the hyphal surface showing dense regions of *PoP* clusters (Figure 4d–f), which was not possible to resolve using confocal microscopy previously.^6,9^ The cross-sectional diameter of the aggregates was ≥50 nm. Going down the length scales, transmission electron microscopy (TEM) of resin-embedded protein–fungal hyphae composite was consistent with STED, validating this finding (see Figure 4g–i). The outer cell wall of the hyphae (Figure 4g) appears to have similar, comparatively low electron density (labeled A) as the hyphae–*PoP* composite (Figure 4i), which demonstrated that surface protein coating did not change significantly. In contrast, there is what appears to be a uniform distribution of electron dense material in the hyphae–*EWP* composite (Figure 4h) supporting the idea of protein coverage of the hyphal outer cell wall.

**Figure 4:**
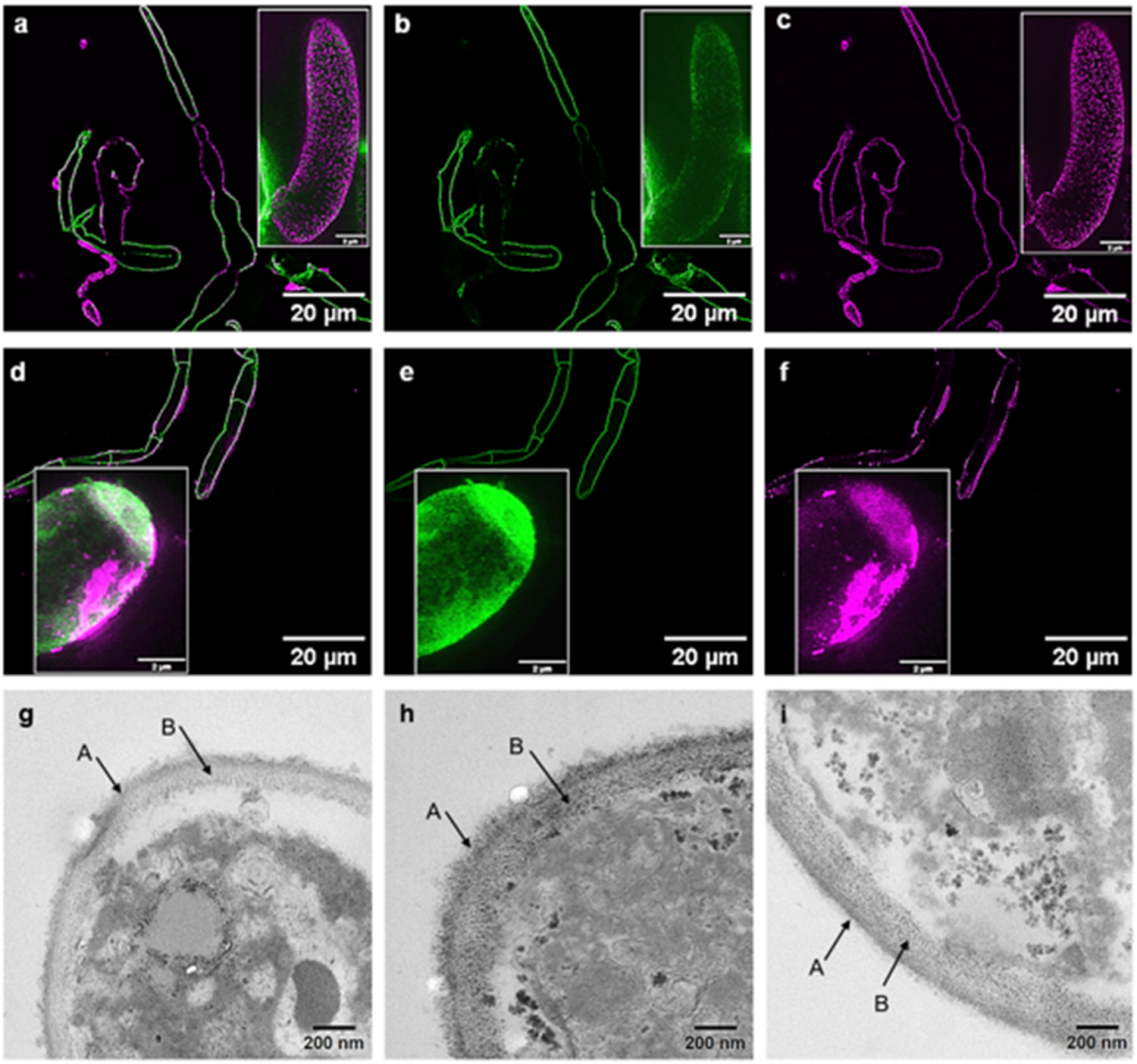
Visualization of protein binding to hyphae surfaces. Deconvolved stimulated emission depletion (STED) maximum intensity projection of six images of fungal hyphae with (a–c) *EWP* and (d–f) *PoP*. Images (a, d) show the full composite image, (b, e) highlight the fungal hyphae and, (c, f) the respective protein binding. Fungal hyphae are labeled with wheat germ agglutinin conjugated to Alexa Fluor 594 shown in green. *PoP* and *EWP* are labeled with antipatatin, and antiovalbumin and secondary antibodies IgG linked to Star Red are shown in pink. Scale bar is 20 and 2 μm for inner images. Transmission electron microscopy (TEM) images of the cell wall of heat-treated Fusarium venenatum showing the morphology of (g) fungal hyphae, (h) fungal hyphae—*EWP* composite, and (i) fungal hyphae—*PoP* composite. Arrows highlight the electron dense (A) and electron transparent (B) areas of the fungal cell wall. Scale bar = 200 nm.

The observed differences in protein aggregation and surface coating behavior can be explained by considering the kinetics of patatin and ovalbumin denaturation. Pots, Gruppen, de Jongh, van Boekel, Walstra, and Voragen^39^ showed that patatin undergoes reversibly partial unfolding at low temperatures ∼28 °C to form a reactive strand with increased exposure of hydrophobic regions and a single thiol group at neutral pH conditions. This loss of structure is also pH-dependent and occurs at pH ≤ 4.5.^39^ Interaction of these exposed hydrophobic regions are dependent on protein concentration, temperature and pH conditions and have been demonstrated to be the main mechanism of patatin–patatin aggregation with the formation of disulfide bonds between thiol groups of a neighboring unfolded molecule playing a minor role.^10,37,39–41^ Therefore, in this particular instance, *PoP*–*PoP* interaction supersedes *PoP–*fungal interaction. Conversely, ovalbumin begins to unfold at ∼76 °C implying much better thermal stability at room temperature and neutral pH conditions.^39,42^ In essence, at room temperature and neutral pH conditions, a solution of ovalbumin is made of mostly globular proteins, while patatin exists as a mixture of globular and partially unfolded proteins, which form the aggregates observed in the STED micrographs.

### Interaction of Protein-Coated Hyphal Surfaces Using SCF-Assisted Calculations

Having discussed the density profiles and spatial organization of proteins at the hyphal surface, it is also useful to calculate the interaction potentials of adsorbed protein layers as a function of pH and background [NaCl]. This would give insight into the binding capability of the two proteins, where a more attractive potential is presumed to indicate better binding. For two flat surfaces, the interaction potentials are calculated as the difference in free energy of the system at each separation distance relative to ‘infinite’ surface separation, here set at a sufficiently large value ≥100*a*_0_. Note, *a*_0_ is the nominal monomer unit size of 0.3 nm, which approximately corresponds to the length of a peptide bond.^11,12,31^ The calculated interaction potential is the sum of the van der Waals and electro-steric interactions.

The interaction potential, *V*_cy_(*h*), between two identical parallel cylinders of radius *R* can be obtained from the SCF-calculated interaction potential between planar surfaces *V*_pl_(*y*) (plotted in Figure S4) using Derjaguin approximation, where $V_{\mathrm{cy}}\left(h\right)=\sqrt{R}\int_{h}^{\infty }\frac{V_{\mathrm{pl}}\left(y\right)}{\sqrt{y-h}}dy$ . Derivation of the corresponding equation for the spheres is commonly highlighted in many articles and books. However, since this is not the case for cylinders which is more representative of the hyphae configuration here, we provide a derivation of the above equation in the method section. With regard to the van der Waals component of the interaction a composite Hamaker constant (*A*_H_) of 5 *k*_B_ *T* for hyphae dispersed in water was assumed. This is a typical value for protein-based particles in water.^43,44^

The net interaction potentials (*U*_TOT_) per unit length of cylinders, as mediated by adsorbed ovalbumin (Figure 5a–c) and patatin (Figure 5d–f), are plotted against surface separation, *h*, between two parallel cylinders of radius *R.* The results are obtained at various pH values and are all expressed in units of *k*_B_ *T*·nm^–1^. The van der Waals component of the interaction is already well-known and is given by the expression $V_{\mathrm{cyl}}\left(r\right)=\frac{-A\sqrt{R}}{24r^{3/2}}$ between the two parallel cylinders, separated by a distance *h* = *r*. We also plot the graphs for this latter, nonprotein-mediated interaction part separately for comparison, as indicated by black dashed lines.

**Figure 5:**
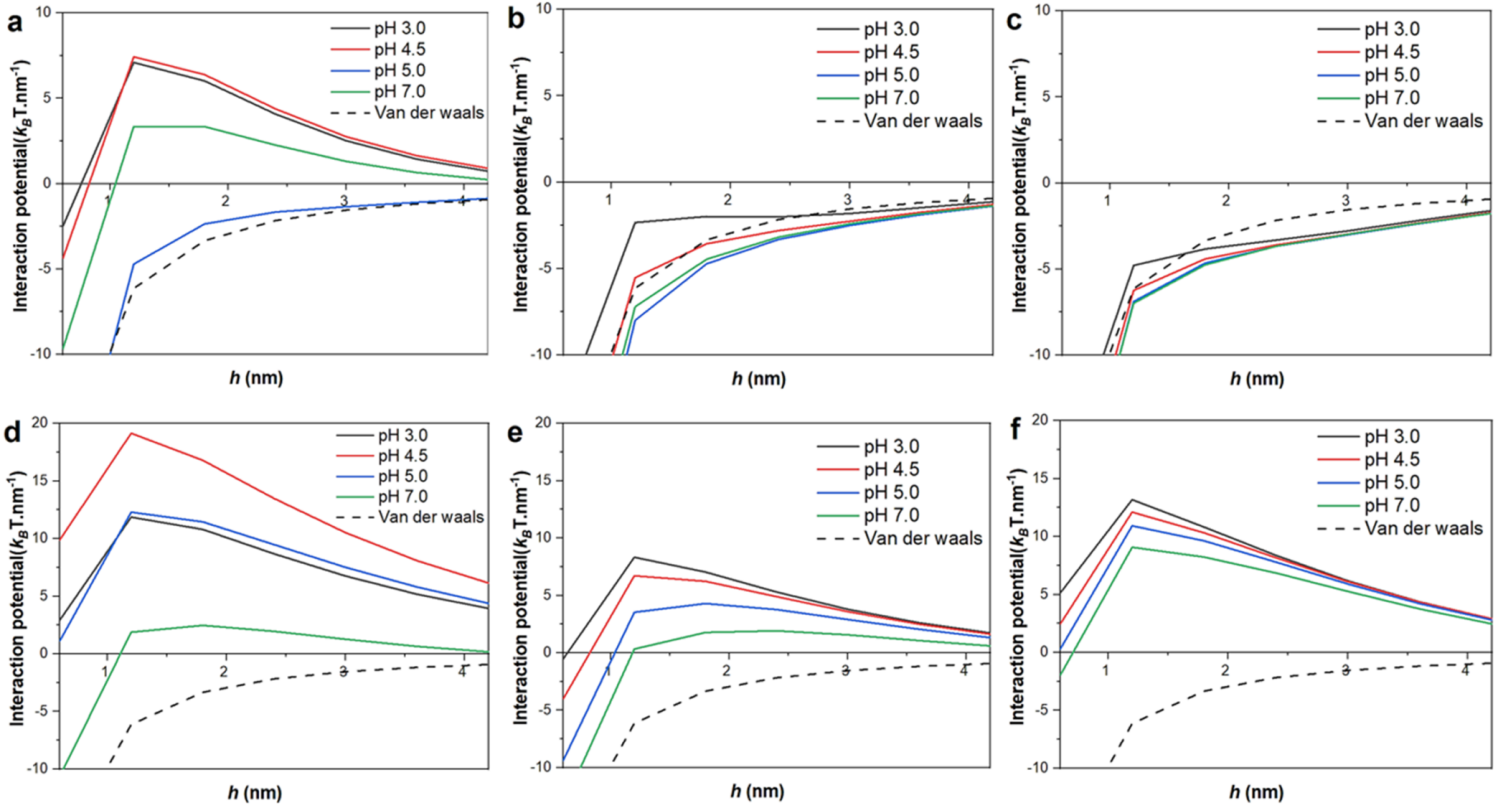
SCF-calculated interaction potential between protein-coated fungal surfaces modeled as weakly hydrophobic cylinders. The interaction potential per unit length between two parallel cylinders (radius, 1.5 μm) arising from adsorbed ovalbumin (a–c) and patatin (d–f) plotted against surface separation between the cylinders, *h*, as a function of pH 3.0 (black solid line), 4.5 (red solid line), 5.0 (blue solid line), and 7.0 (green solid line) with van der Waals interaction (dashed line) included for comparison. Changes to interaction potential arising from alteration of background salt volume fraction 0.001 (a, d), 0.01 (b, e), and 0.05 (c, f) are demonstrated for both proteins.

The interaction potential with ovalbumin at *ϕ*_*s*_ = 0.001, approximately implying [NaCl] = 10 mM (Figure 5a), varies significantly with pH. At pH values away from the pI (∼pH 5.0, blue curve), i.e., pH 3.0 (black), pH 4.5 (red), and pH 7.0 (green), there is a significant positive (i.e., repulsion) interaction at a separation *h* = 1 nm. However, this repulsive potential drops off sharply with increasing *h*, so that by *h* = 5 nm, it is essentially zero (see Figure S5 for the expanded graphs). Close to the pI, there is little or no electrostatic repulsion, and therefore, one expects that the total interaction *U*_TOT_ will consist almost entirely of the steric and van der Waals interaction forces. Comparing this *U*_TOT_ to its van der Waals component part (dashed line) at pH 5.0 indicates that the steric interaction is minute and insufficient to overcome the attractive van der Waals forces between the surfaces. Moving away from the pI to pH 4.5 (red curve), there is a significant increase in the repulsion, decreasing slightly with further reduction in pH to pH 3.0.

Increasing *ϕ*_*s*_ to 0.01 ≈ [NaCl] = 100 mM (Figure 5b) screens the protein charge, eliminating the electrostatic repulsion between the protein layers and results in similar *U*_TOT_ versus *h* curves at all pH that do not differ significantly from the purely attractive van der Waals component. Further increase in *ϕ*_*s*_ to 0.05 ≈ [NaCl] = 500 mM (Figure 5c) leads to a slight increase in attractive potential due to increased protein adsorption, as described earlier. The responses to pH and salt clearly suggest that electrostatic interaction is the main component of the repulsive interaction for ovalbumin-coated fungal hyphae, with steric interactions playing a less dominant role. This is consistent with the previous macroscopic reports on the rheology of the composites as a function of pH and salt.^6^ Also, the negative interaction potential observed at the highest [NaCl] supports the idea of the existence of attractive bridging interactions between the surfaces at the close surface separations. This occurs with proteins having many short segments of hydrophobic and hydrophilic residues in their backbone that are then more prone to simultaneous adsorption on two adjacent surfaces which then tends to cause bridging flocculation. This attractive bridging under most pH and salt conditions explains how ovalbumin, and by extension *EWP*, provides superior binding properties between fungal hyphae surfaces.

Patatin shows significantly different behavior as compared to ovalbumin. At *ϕ*_*s*_ of 0.001 ([NaCl] ∼ 10 mM) (Figure 5d), there is a repulsive interaction potential at all pH conditions. This is the case even close to the protein pI ∼ pH 5.0, indicating the presence of non-Coulombic repulsive forces large enough to overcome the attractive van der Waals interactions. As shown in Figure 3d–f, the adsorbed configuration of patatin can clearly account for this in terms of steric repulsion due to the protruding hydrophilic parts of the chains. There is significant variability of the repulsive interaction with pH, but nonetheless, it remains positive with the lowest level of repulsion observed at neutral pH 7.0 conditions.

Screening patatin’s charge by increasing *ϕ*_*s*_ to 0.01 ≈ [NaCl] = 100 mM (Figure 5e) reduces the positive (repulsive) interaction potential, with further increase to *ϕ*_*s*_ to 0.05 equiv to [NaCl] = 500 mM (Figure 5f), leading to even further reduction in the repulsion, with less variation with pH. This trend with increasing [NaCl] is again due to increased screening of the charged extended chains (decreasing mutual repulsion between like-charged adjacent chains) but allowing for increased protein adsorption at higher [NaCl]. Thus, unlike ovalbumin- coated surfaces, where the repulsion is almost entirely controlled via electrostatics, patatin appears to mediate both steric and electrostatic components, with the former playing a more dominant role. More crucial is the absence of an overall attractive potential between patatin-coated surfaces, which indicates *PoP* will be a less efficient binder of the fungal hyphae in real composites.

### Experimental Validation of Interaction Forces via AFM-Colloidal Probe Force Spectroscopy

Finally, we measured interaction potentials of *EWP* and *PoP* via AFM to validate the SCF predictions in Figure 6 (see Figure S6 for the expanded force-distance curves and curves between clean silicon surfaces). About 1.0 wt % protein solutions with very little salt present estimated to be <1.0 mM were left to adsorb onto silicon wafer and SiO_2_ colloidal probe of radius 1 μm. Adsorbed protein layers were then adjusted to pH 7.0, 5.0, 4.5, and 3.0 conditions and force–distance curves measurement in MQ-water.

**Figure 6:**
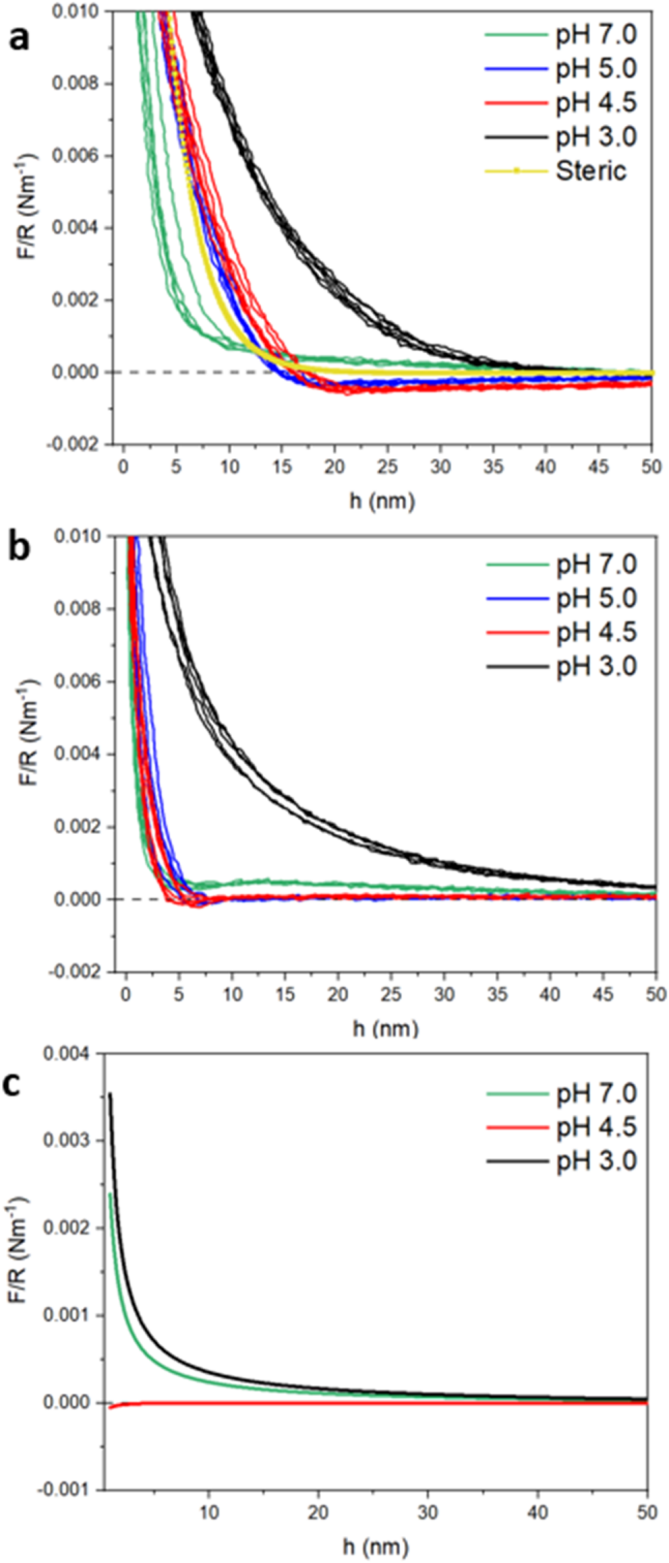
Experimental AFM force versus separation curves, plotted as force/radius (*F*/*R*) versus probe-sample surface separation (*h*) of (**a**) *EWP*- and (**b**) *PoP-*coated silica colloidal probes and silicon wafer obtained at pH 3.0 (black solid line), pH 4.5 (red solid line), pH 5.0 (blue solid line), pH 7.0 (green solid line), and predicted steric force contribution (yellow). (**c**) Calculated DLVO *F*/*R* versus *h* of the ovalbumin. Multiple data sets from three replicates on the same systems (*n* = 3 × 2) are shown to indicate the typical reproducibility.

In agreement with the SCF-calculated interaction forces, *EWP* (Figure 6a) at pH 4.5 gave the highest surface attraction (negative force), which increased as the surfaces approached until *h* ∼ 20 nm. This is the pI of ovalbumin (the main constituent of *EWP*) where the protein is minimally charged, and therefore, there should be minimal electrostatic repulsion between the protein layers. A slight increase to pH 5.0 introduces more surface charge and therefore repulsion, which reduces its attractive surface interaction, but it stays negative at *h* ≥ 17 nm. Further increases in protein surface charge at pH 7.0 and 3.0 overcome the attractive forces, leading to an overall repulsive interaction force between *EWP* layers at all *h*. The strong short-range repulsive force is probably steric in nature and will be a consequence of the combination of all the proteins that make up *EWP*, as well as any aggregates that they have formed.^45,46^ However, despite these complications with *EWP*, the overall trends earlier predicted by the SCF calculations is validated—that ovalbumin does indeed dominate the overall interaction forces with *EWP*, which is largely Coulombic in nature. In contrast to *EWP*, the interaction forces between *PoP-*coated surfaces as a function of *h* remain positive, i.e., repulsive, at all measured pH conditions (Figure 6b). The lowest repulsive interaction forces are measured at pH 4.5 and pH 5.0, which are within the range of the pI of patatin (pH 4.5–5.2). This corroborates the SCF calculations and points out that steric repulsion is indeed the main controlling interaction mechanism of *PoP*.

As a first attempt to explain the data in Figure 6a and b quantitatively, we used a DLVO model to predict the combined effects of the electrostatic repulsion between the surfaces and the attractive van der Waals interaction. We used the model of Carnie, Chan, and Gunning^47^ to describe the electrostatic interaction between a sphere and a flat (equivalent to a sphere of extremely large radius in their model), using the assumption of a constant surface charge, which is probably the most appropriate for protein-covered surfaces in this medium. We also assumed that the surfaces were coated with enough protein so that the surface potential of both surfaces was the same and equal to the measured values of the ζ-potential (see Table S1) for the proteins at the appropriate pH, i.e., −23.9, −3.24, and +28.87 mV for pH 7.0, 4.5, and 3.0, respectively. Again, this seems to be a reasonable assumption, for example, when the ζ-potential of protein-stabilized oil (emulsion) droplets is compared with the ζ-potential of the proteins themselves.^48–50^

The van der Waals interaction for a silica sphere interacting with a silicon substrate was calculated using the model of Wang, Wang, Hampton, and Nguyen,^30^ with a 0.5 nm offset in the start of the electrostatic repulsion to account for the short silica “hairs” that are usually present on SiO_2_ surfaces. We included this in our calculations, shown in Figure 6c, although this makes very little difference to the curves or the overall conclusion—that the measured repulsion for *EWP* far exceeds the predicted repulsion at short *h* at pH 3.0 and 7.0. At pH 4.5, close to the pI of ovalbumin, the predicted and measured forces are much closer for *h* > 20 nm, in fact both are close to zero net *F*/*R*, as expected when the net charge on ovalbumin is zero, but for *h* < 20 nm, the measured forces become increasingly repulsive as *h* decreases, while the predicted *F*/*R* start to become slightly negative (i.e., attractive), as expected for this low surface charged scenario (ζ-potential = −3.2 mV).

Note that the assumed ionic strength is unlikely to be less than 10^–4^ M in all cases, plus the constant surface charge model gives a larger repulsion than a self-regulating (constant surface potential model), so that these predicted curves probably represent the *maximum* in any kind of electrostatic repulsive contribution, as long as the high underlying charge on the SiO_2_ is masked by that of the adsorbed protein.^30,47^ The most likely explanation of the larger repulsive force at short-range is therefore a steric contribution from adsorbed *EWP*. There are no precise analytical equations for this steric force for adsorbed polyelectrolytes (hence the use of the SCF model earlier) but the simplest approximate model is to add in an exponentially decaying force of the form:

8 $$F_{s}=S\cdot \exp\left(-\frac{h}{L}\right)\left(x\right)$$

where *L* is a factor representative of the adsorbed polymer dimensions (measured normal to the surface), often taken as the radius of gyration of the adsorbed polymer in the bulk as a first approximation. *S* is an arbitrary prefactor indicating the high steric repulsion at “zero” separation. Taking *L* = 3 nm for ovalbumin and *S* = 3 × 10^4^, *k*_B_ *T* gives the predicted steric contribution indicated by the yellow line in Figure 6a. It is seen that this choice gives an interaction that is very close to the experimental data for pH 4.5, when the net charge on ovalbumin is almost zero, i.e., the electrostatic repulsion is minimal. The choice of *S* is purely arbitrary, but *L* = 3 nm is representative of the SCF-predicted length scales of penetration of the ovalbumin chain into the bulk (see Figure 3). This simple representation of a steric force based on eq 8 therefore substantiates the assumption that most of the discrepancy between the measured and DLVO-predicted results is due to the lack of an appropriate steric contribution, further confirming the SCF data reinforcing the importance of Coulombic forces in *EWP* unlike *PoP*.

## Conclusions

This work advanced our understanding of the hyphal-binding mechanism of plant and animal proteins in composite systems, with deep insights into the most desirable properties of alternative, plant-based hyphae binders. In summary, our results highlight the uniform hyphal coating properties of ovalbumin and *EWP* and the resulting attractive surface potential, which explain their better performance as hyphal binders. Conversely, the susceptibility of patatin and *PoP* to self-aggregation results in nonuniform attachment of protein aggregates to the hyphal surface. Coupled with the greater influence of steric-induced surface repulsive potential, *PoP* delivers an inferior hypha binding ability relative to that of *EWP*. Therefore, an attempt to replicate the behavior of *EWP* can be streamlined by evaluating alternative protein(s) that offer uniform hyphal coating with minimal self-aggregation and repulsive interaction potential. It should be noted that the model cylinders and experimental silicon-SiO_2_ surfaces do not replicate the morphology and chemical complexity of the actual fungal hyphae; therefore, more research into this area is necessary.

In terms of practical food applications, this research demonstrates that the choice of alternative proteins to replicate the behavior and performance of animal proteins like *EWP* as binders of composite food systems should go beyond similarities in protein physicochemical properties like isoelectric point, solubility, and ratio of hydrophobic to hydrophilic amino acids, where *PoP* appears to be identical. Instead, significant consideration should be given to the underlying protein adsorption and adsorbed layer interaction behavior, which can be appraised via theoretical calculations like the self-consistent field theory utilized here.

## Supplementary Material



## Notes

The Supporting Information file contains six figures (Figures S1–S6) and two tables (**Table S1** and **S2**); **Figure S1** shows the theoretical protein charge calculation as a function of the pH of ovalbumin and patatin used in our model; **Figure S2** shows SCF-calculated density profiles (volume fraction (ϕ) plotted against the perpendicular distance from the hyphal surface) of ovalbumin and patatin as a function of pH and background NaCl volume fraction; **Figure S3** shows SCF-predicted adsorption isotherms of ovalbumin and patatin onto weakly hydrophobic cylinder surfaces modeling the fungal hyphae surfaces as a function of pH and background NaCl volume fraction; **Figure S4** shows SCF-calculated interaction potential (obtained as the difference in free energy as a function of surface separation distance in units of *a*_0_) of ovalbumin and patatin adsorbed on planar surfaces as a function of pH and background NaCl volume fraction; **Figure S5** shows the expanded graph of SCF-calculated interaction potentials of ovalbumin and patatin adsorbed to two weakly hydrophobic parallel cylinders modeling the fungal hyphae as a function of pH and background NaCl volume fraction; **Figure S6** shows AFM force–distance curves as a function of pH of protein-coated silicon and silicon dioxide surfaces, shown over a wider range of separation distance and force range than in Figure 6; **Table S1** shows the physicochemical characteristics (ζ-potential, electrophoretic mobility, hydrodynamic diameter, and polydispersity index) of *EWP* and *PoP* prepared at 0.3 wt %; **and Table S2** shows ζ-potential and electrophoretic mobility of crushed fungal hyphae dispersion as a function of pH (PDF)
