# Supplementary material for: Unravelling Protein–Fungal Hyphae Interactions at the Nanoscale
Source: ACS Appl Mater Interfaces. 2025 Apr 17;17(20):30212–24. doi: 10.1021/acsami.5c01064 (PMC12100587; doi:10.1021/acsami.5c01064)
Supplement: Supplementary file 1 [file am5c01064_si_001.pdf]

Supporting Information

Unravelling protein-fungal hyphae interactions at the nanoscale

Mary C. Okeudo-Cogan[1, 2], Brent S. Murray[2]**, Rammile Ettelaie[2], Simon D. Connell[3], Michelle Peckham[4], Ruth E. Hughes[4], Martin J. G. Fuller[4], Stewart J. Radford[5], and Anwesha Sarkar[2]*

[1]School of Chemical and Process Engineering, University of Leeds, Leeds, LS2 9JT, UK

[2]Food Colloids and Bioprocessing Group, School of Food Science and Nutrition, University of Leeds, Leeds, LS2 9JT, UK

[3]School of Physics and Astronomy, University of Leeds, Leeds, LS2 9JT, UK

[4]Faculty of Biological Sciences, University of Leeds, Leeds, LS2 9JT, UK

[5]Quorn Foods, Station Road, Stokesley, North Yorkshire, TS9 7AB, UK

Corresponding authors:

**Prof. Brent S. Murray

E-mail address: B.S.Murray@leeds.ac.uk (B.S. Murray).

*Prof. Anwesha Sarkar

E-mail address: A.Sarkar@leeds.ac.uk (A. Sarkar).

Food Colloids and Bioprocessing Group,

School of Food Science and Nutrition,

University of Leeds, Leeds LS2 9JT, UK.

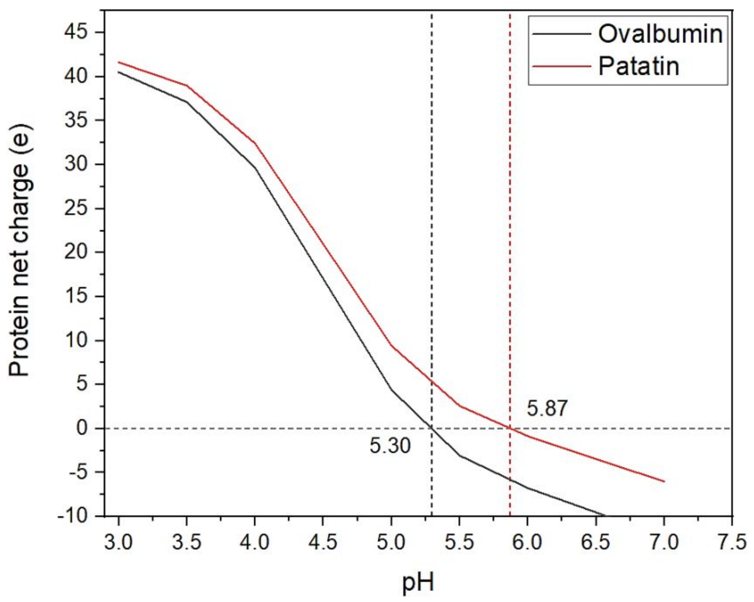

Figure S1. Theoretical protein charge calculation as a function of pH. Variation of electrostatic charge (*e*) calculated from the ionisable groups (groups 3 to 6) of ovalbumin (black solid line) and patatin (red solid line) amino acid residues as a function of pH. Vertical dashed lines highlight the isoelectric point of both the model proteins. Protein primary sequence was obtained from the protein database UniProt.[1-3]

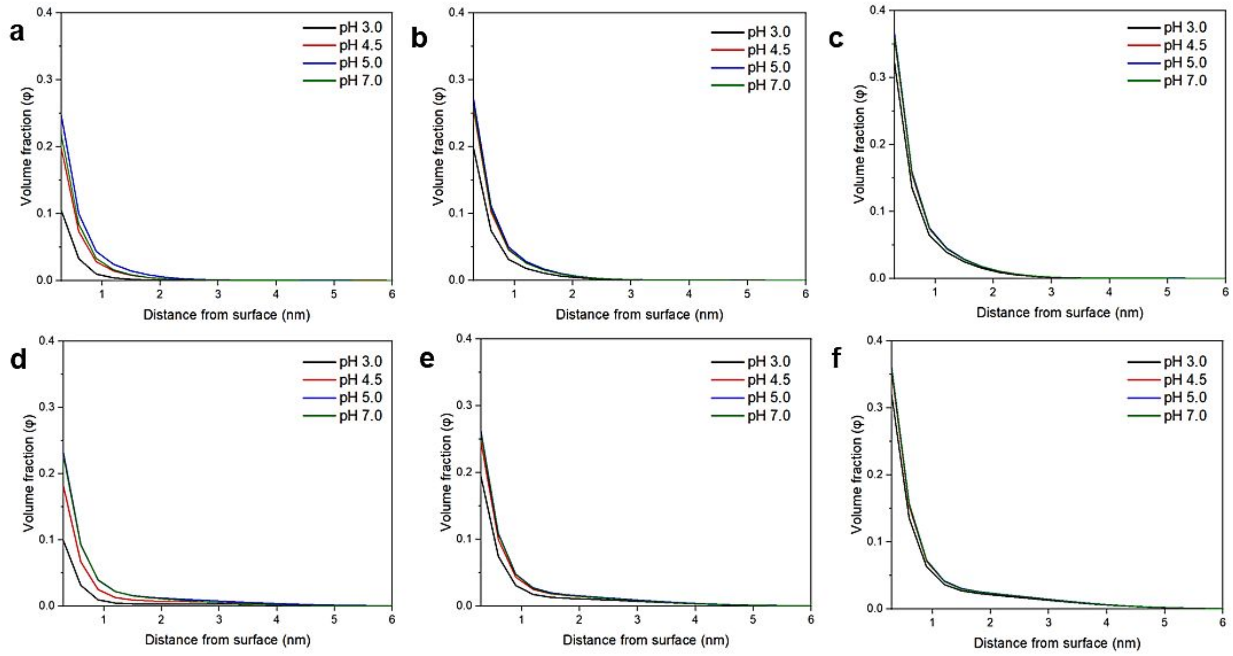

Figure S2. SCF calculated density profiles of proteins on weakly hydrophobic surfaces modelling the fungal hyphae. Volume fraction (ϕ) of ovalbumin (**a-c**) versus patatin (**d-f**) plotted against perpendicular distance from the hyphal surface as a function of pH 3.0 (black solid line), 4.5 (red solid line), 5.0 (blue solid line), 7.0 (green solid line) and background NaCl volume fraction 0.001 (**a, d**), 0.01 (**b, e**) and 0.05 (**c, f**).

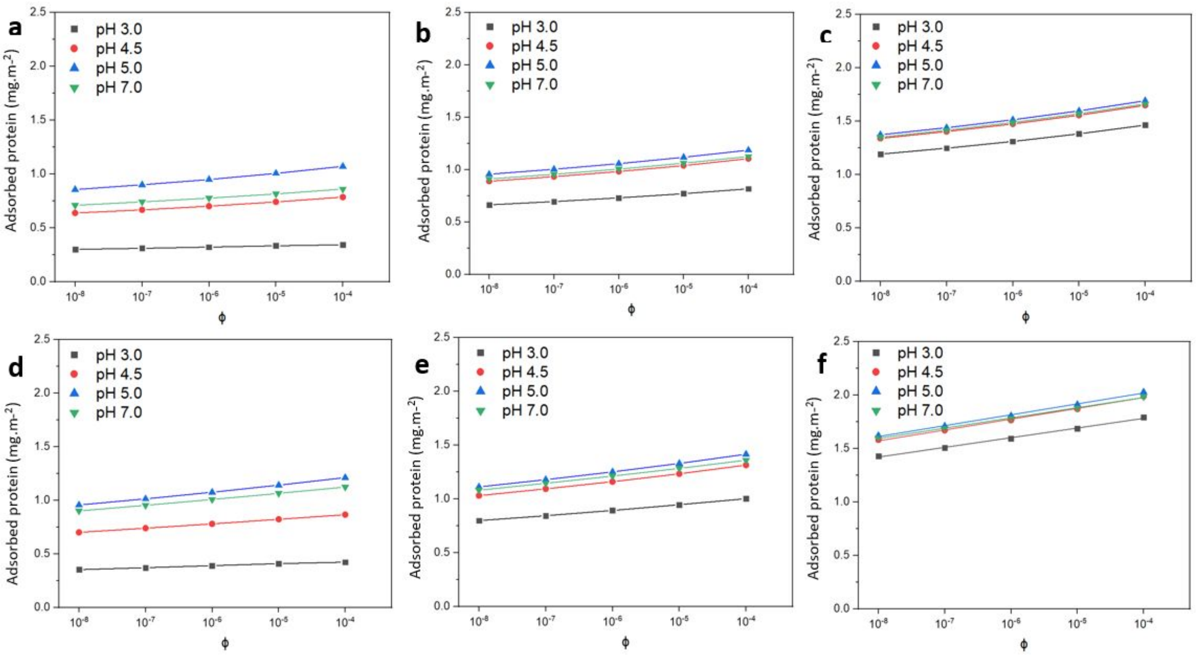

Figure S3. SCF predicted adsorption isotherms of proteins onto weakly hydrophobic cylinder surfaces modelling the fungal hyphae surfaces. Predicted adsorption isotherms of ovalbumin (**a-c**) and patatin (**d-f**) onto a hydrophobic surface: Γ = adsorbed amount, ϕ_p = bulk protein concentration from 10^{-8} to 10^{-4} as a function of pH 3.0 (black solid line), 4.5 (red solid line), 5.0 (blue solid line), 7.0 (green solid line) and background salt volume fraction 0.001 (**a, d**), 0.01 (**b, e**) and 0.05 (**c, f**). An average molecular weight of 42,881 Da for both polypeptide chains (ovalbumin and patatin) was used to convert the total adsorbed amount (Γ) into units of mg m^{-2}.[4, 5]

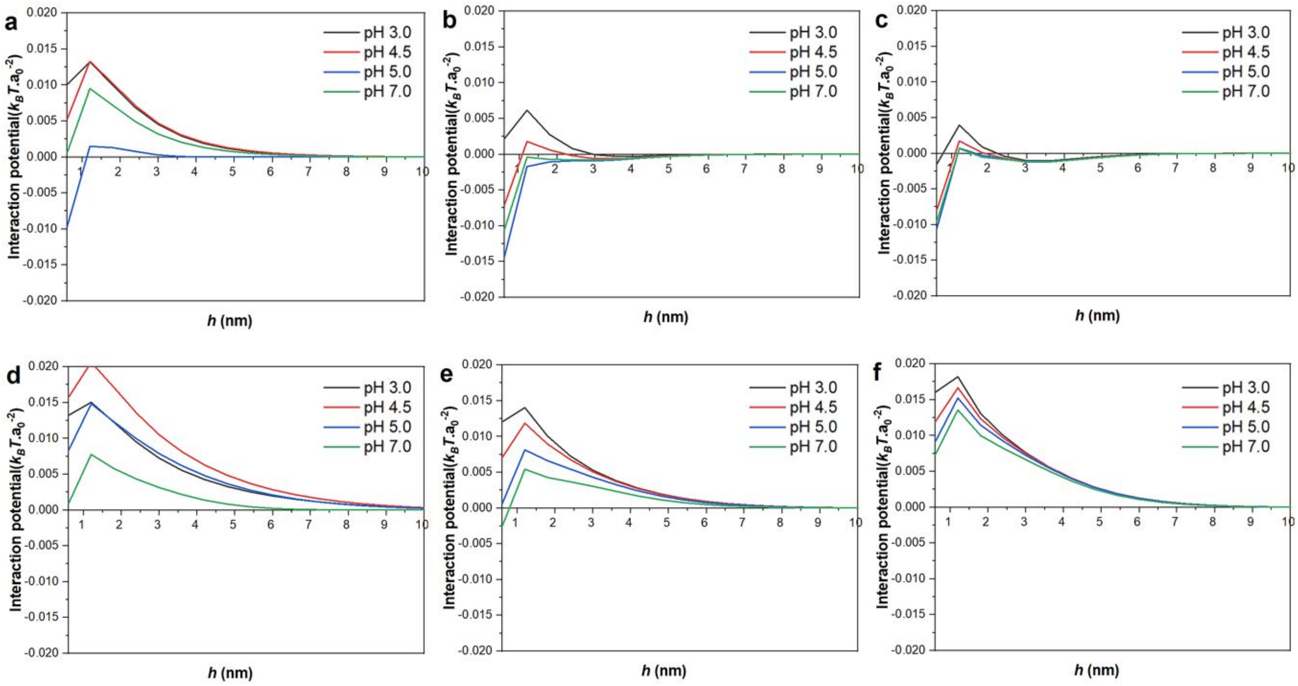

Figure S4. SCF calculated interaction potential of proteins adsorbed on planar surfaces. The interaction potential between two flat planar surfaces were obtained as the difference in free energy as a function of surface separation distance in units of a_0 arising from adsorbed ovalbumin (**a-c**) and patatin (**d-f**) plotted against separation distance, *h,* at various values of pH 3.0 (black solid line), 4.5 (red solid line), 5.0 (blue solid line), 7.0 (green solid line). Changes to interaction potential as a function of salt volume fraction 0.001 (**a, d**), 0.01 (**b, e**) and 0.05 (**c, f**) is demonstrated for both proteins.

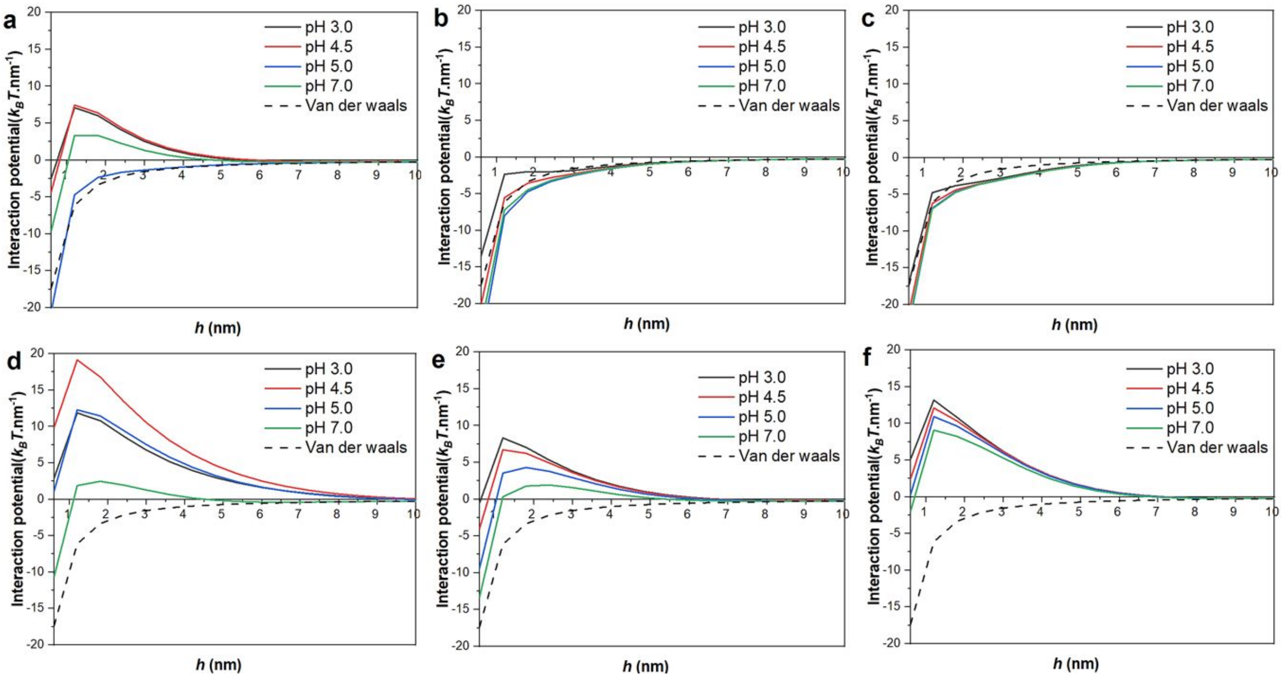

Figure S5. SCF calculated interaction potentials of proteins adsorbed to two weakly hydrophobic parallel cylinders modelling the fungal hyphae The interaction potential between two parallel cylinders (radius, 1.5 μm) arising from adsorbed ovalbumin (**a-c**) and patatin (**d-f**) plotted against separation distance of *h*, at various values of pH 3.0 (black solid line), 4.5 (red solid line), 5.0 (blue solid line), 7.0 (green solid line) with attractive van der Waals component of the interaction (dashed line) included for comparison. Changes to interaction potential as a function of salt volume fraction 0.001 (**a, d**), 0.01 (**b, e**) and 0.05 (**c, f**) is demonstrated for both proteins.

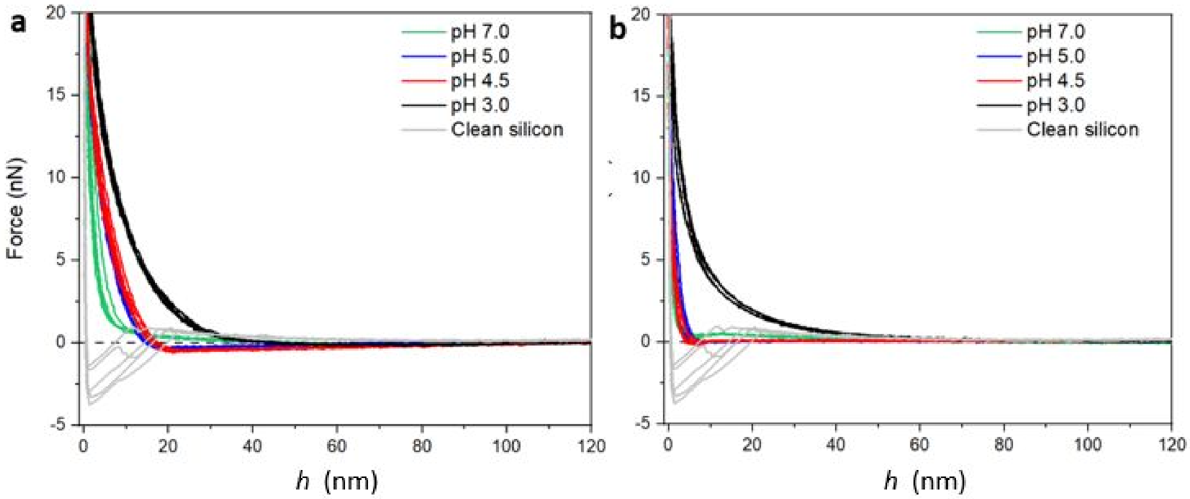

Figure S6. AFM force-distance curves of protein coated silicon and silicon dioxide surfaces, shown over a wider range of separation distance and force range than in Figure 6. Force versus separation distance (*h*) approach curves between (**a**) *EWP* and (**b**) *PoP* coated-colloidal probes and silicon wafer at pH 3.0 (black solid line), 4.5 (red solid line), 5.0 (blue solid line), 7.0 (green solid line). AFM Force – distance curves between clean SiO_2 colloidal probes and clean silicon wafer surface (grey solid line) in Milli-Q water (n =3 x 2).

Table S1. Physicochemical characteristics of *EWP* and *PoP*. *EWP* and *PoP* in Milli Q water prepared at a concentration of 0.3 wt. % and then passed through a 0.2 μm filter before pH adjustment. Columns represent means ± standard deviation, n = 3 × 3.

Egg white protein				
pH	ζ-potential (mV)	U_E (μmcm/Vs)	d_H (nm)	PDI
7.0	-23.9 ± 0.26	-1.87 ± 0.02	830.77 ± 24.56	0.89 ± 0.1
5.0	-3.3 ± 0.07	-0.26 ± 0.01	2743.67 ± 65.21	0.43 ± 0.04
4.5	3.24 ± 0.07	0.25 ± 0.01	462.67 ± 11.55	1 ± 0
3.0	28.87 ± 0.12	2.26 ± 0.01	566.77 ± 5.3	0.66 ± 0.01
Potato protein				
pH	ζ-potential (mV)	U_E (μmcm/Vs)	d_H (nm)	PDI
7.0	-28.03 ± 0.42	-2.2 ± 0.03	24.51 ± 0.34	0.77 ± 0.02
5.0	-6.48 ± 0.17	-0.51 ± 0.01	2701.33 ± 68.38	0.41 ± 0.1
4.5	5.86 ± 0.21	0.47 ± 0.02	2895.33 ± 75.08	0.32 ± 0.03
3.0	33.77 ± 0.21	2.65 ± 0.02	71.92 ± 1.74	0.51 ± 0.03

U_E – electrophoretic mobility, d_H – hydrodynamic diameter, PDI – polydispersity index.

Table S2. ζ-potential and electrophoretic mobility shown as mean ± standard deviation of fungal hyphae dispersion as a function of pH, n = 3 x 3. The fungal paste was frozen in liquid nitrogen and crushed repeated until a fine paste was obtained. Washed hyphal particles were filtered through a 0.2 μm filter before pH adjustment. Hyphal dispersions were at measured at ~ 0.5 g/L.

pH	ζ-potential (mV)	U_E (μm.cm/Vs)
3.0	-13.53 ± 0.32[a]	-1.06 ± 0.03[a]
4.0	-16.83 ± 1.67[b]	-1.32 ± 0.13[b]
5.0	-21.7 ± 0.62[c]	-1.7 ± 0.05[c]
5.5	-24.53 ± 1.27[d]	-1.92 ± 0.1[d]
6.0	-27.17 ± 0.64[de]	-2.13 ± 0.05[de]
6.5	-29.2 ± 0.95[e]	-2.29 ± 0.07[e]
7.0	-33.97 ± 0.4[f]	-2.66 ± 0.03[f]

Columns with the same letter of the alphabet are not significantly different at $p \leq 0.05$. U_E – electrophoretic mobility.

References

(1) Thompson, E.; Fisher, W. A Correction and Extension of the Acetylated Amino Terminal Sequence of Ovalbumin. *Australian Journal of Biological Sciences* **1978**, *31* (5), 443-446. DOI: https://doi.org/10.1071/BI9780443.

(2) Henderson, J. Y.; Moir, A. J. G.; Fothergill, L. A.; Fothergill, J. E. Sequences of Sixteen Phosphoserine Peptides from Ovalbumins of Eight Species. *European Journal of Biochemistry* **1981**, *114* (2), 439-450. DOI: https://doi.org/10.1111/j.1432-1033.1981.tb05165.x (acccessed 2024/09/26).

(3) Bevan, M.; Barker, R.; Goldsbrough, A.; Jarvis, M.; Kavanagh, T.; Iturriaga, G. The Structure and Transcription Start Site of a Major Potato Tuber Protein Gene. *Nucleic Acids Res* **1986**, *14* (11), 4625-4638. DOI: 10.1093/nar/14.11.4625 From NLM.

(4) Racusen, D.; Weller, D. L. Molecular Weight of Patatin, a Major Potato Tuber Protein. *Journal of Food Biochemistry* **1984**, *8* (2), 103-107. DOI: https://doi.org/10.1111/j.1745-4514.1984.tb00318.x (acccessed 2024/08/05).

(5) Nisbet, A. D.; Saundry, R. H.; Moir, A. J. G.; Fothergill, L. A.; Fothergill, J. E. The Complete Amino-Acid Sequence of Hen Ovalbumin. *European Journal of Biochemistry* **1981**, *115* (2), 335-345. DOI: https://doi.org/10.1111/j.1432-1033.1981.tb05243.x (acccessed 2024/08/05).
